# Comparison of T1 mapping techniques for ECV quantification. Histological validation and reproducibility of ShMOLLI versus multibreath-hold T1 quantification equilibrium contrast CMR

**DOI:** 10.1186/1532-429X-14-88

**Published:** 2012-12-28

**Authors:** Marianna Fontana, Steve K White, Sanjay M Banypersad, Daniel M Sado, Viviana Maestrini, Andrew S Flett, Stefan K Piechnik, Stefan Neubauer, Neil Roberts, James C Moon

**Affiliations:** 1 The Heart Hospital, 16-18 Westmoreland Street, London, W1G 8PH, United Kingdom; 2 Institute of Cardiovascular Science, University College London, London, WC1E 6BT, United Kingdom; 3 Department of Cardiovascular Medicine Oxford Centre for Clinical Magnetic Resonance Research, University of Oxford, Oxford, OX3 9DU, United Kingdom

**Keywords:** Interstitial space, Fibrosis, CMR

## Abstract

**Background:**

Myocardial extracellular volume (ECV) is elevated in fibrosis or infiltration and can be quantified by measuring the haematocrit with pre and post contrast T1 at sufficient contrast equilibrium. Equilibrium CMR (EQ-CMR), using a bolus-infusion protocol, has been shown to provide robust measurements of ECV using a multibreath-hold T1 pulse sequence. Newer, faster sequences for T1 mapping promise whole heart coverage and improved clinical utility, but have not been validated.

**Methods:**

Multibreathhold T1 quantification with heart rate correction and single breath-hold T1 mapping using Shortened Modified Look-Locker Inversion recovery (ShMOLLI) were used in equilibrium contrast CMR to generate ECV values and compared in 3 ways.

Firstly, both techniques were compared in a spectrum of disease with variable ECV expansion (n=100, 50 healthy volunteers, 12 patients with hypertrophic cardiomyopathy, 18 with severe aortic stenosis, 20 with amyloid). Secondly, both techniques were correlated to human histological collagen volume fraction (CVF%, n=18, severe aortic stenosis biopsies). Thirdly, an assessment of test:retest reproducibility of the 2 CMR techniques was performed 1 week apart in individuals with widely different ECVs (n=10 healthy volunteers, n=7 amyloid patients).

**Results:**

More patients were able to perform ShMOLLI than the multibreath-hold technique (6% unable to breath-hold). ECV calculated by multibreath-hold T1 and ShMOLLI showed strong correlation (r^2^=0.892), little bias (bias -2.2%, 95%CI -8.9% to 4.6%) and good agreement (ICC 0.922, range 0.802 to 0.961, p<0.0001). ECV correlated with histological CVF% by multibreath-hold ECV (r^2^= 0.589) but better by ShMOLLI ECV (r^2^= 0.685). Inter-study reproducibility demonstrated that ShMOLLI ECV trended towards greater reproducibility than the multibreath-hold ECV, although this did not reach statistical significance (95%CI -4.9% to 5.4% versus 95%CI -6.4% to 7.3% respectively, p=0.21).

**Conclusions:**

ECV quantification by single breath-hold ShMOLLI T1 mapping can measure ECV by EQ-CMR across the spectrum of interstitial expansion. It is procedurally better tolerated, slightly more reproducible and better correlates with histology compared to the older multibreath-hold FLASH techniques.

## Background

The myocardial extracellular space is expanded by focal fibrosis [1], diffuse fibrosis [2-6] or infiltration, such as amyloidosis [7]. It can be measured non-invasively by cardiovascular magnetic resonance (CMR) using pre and post contrast T1 relaxation times of blood and myocardium (the latter at sufficient contrast equilibrium) with correction for the blood volume of distribution via the haematocrit [1,2,8]. A number of T1 measurement techniques exist including multibreath-hold techniques such fast low angle single shot inversion recovery (“multibreath-hold FLASH-IR”). Here the sequence is performed at increasing inversion times to generate T1 recovery curves and heart rate correction [9,10]. Newer, faster sequences such as MOLLI (Modified Look-Locker Inversion recovery) [11] perform IR measurements in a single breath-hold. A recent evolution of MOLLI, the Shortened-MOLLI (ShMOLLI) [12] improves clinical utility with a shorter breath-hold and immediate map reconstruction directly on the scanner. ECV measurements have been performed and validated using MOLLI for bolus-only protocols. Equilibrium contrast CMR (EQ-CMR) with single breath-hold sequences has not yet been validated for ECV assessment. ECV mapping with such sequences would be a significant technical advance, being easier for patients with shorter breath-holds (either shorter scans or whole heart coverage) and easier quantification.

We hypothesised that ECV mapping using ShMOLLI would be superior to the multibreath-hold FLASH-IR technique. This was assessed in three ways: firstly, to determine any bias in ECV between the two techniques. Secondly, we compared both CMR techniques with histological collagen volume fraction (CVF%). Finally, we assessed the reproducibility of both CMR techniques. For equivalence of contrast conditions between the two techniques, we used the primed infusion technique, equilibrium contrast CMR.

## Methods

### CMR protocol

**Figure 1:**
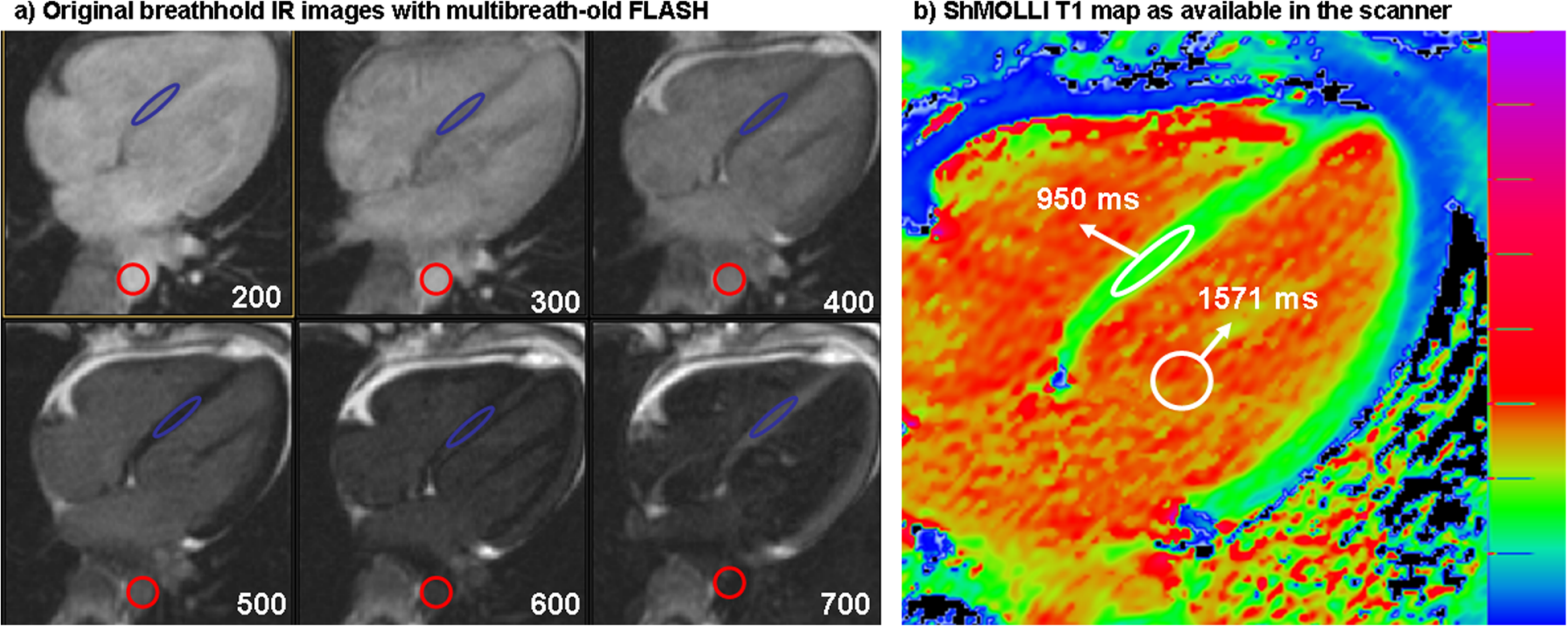
**Pre contrast T1 measurement example.** Left multiple panels: The multibreath-hold T1 measurement uses up to 7 CMR acquisitions with linearly increasing inversion time. T1 value is reconstructed from fitting average signal intensity from individual image intensity averages. Right single panel: ShMOLLI technique generates T1 map directly on the scanner console using pixelwise calculations based on a single breath-hold experiment. ShMOLLI T1 is calculated as average of all pixels from a region of interest drawn in the myocardium.

The research received approval from the local research ethics committee and all participants provided written informed consent. EQ-CMR was performed as described previously [9]. CMR was performed on a 1.5T magnet (Avanto, Siemens Medical Solutions). Within a standard clinical scan (pilots, transverse white and black blood images, volumes, and LGE imaging) T1 measurement pre-contrast was performed using (a) FLASH-IR at increasing inversion times from 140 to 800 ms (or 900 ms if patient heart rate permitted), “multibreath-hold technique”, Figure 1a and (b) ShMOLLI T1 mapping “single breath-hold technique”, Figure 1b. After a bolus of Gadoterate meglumine, (0.1 mmol/kg, gadolinium-DOTA, marketed as Dotarem © Guerbet S.A. France) and standard LGE imaging, at 15-minute post bolus, an infusion at a rate of 0.0011 mmol/kg/min contrast (equivalent to 0.1 mmol/kg over 90 minutes) was given. The patient was typically removed from the scanner at this time. At between 45 minutes and 80 minutes post bolus, the patient was returned to the scanner, still with the infusion, and the T1 measurement repeated using both multi and single breath-hold techniques. Separate regions of interest (ROIs) were placed in all available images and recovery curve was reconstructed by fitting the relaxation formula to ROI averages. Heart rate correction was used for the multibreath-hold technique [9]. In the ShMOLLI sequence, T1 maps were generated using previously published algorithm [12]. A single ROI was drawn directly in each T1 map at the same location as the multibreath-hold technique and T1 averaged between all pixels (Figure 1b). A haematocrit was taken in all subjects. The ECV was calculated with each method as Myocardial ECV = (1-haematocrit) × (ΔR1_myocardium/_ΔR1_blood_) [1]. T1 was measured in the basal to mid septum avoiding areas of late gadolinium enhancement, except in myocardial infarction (where the infarct zone was assessed) and amyloid (where the regions was drawn irrespective of the ill-defined presence/absence of LGE). The blood T1 was assessed in the descending aorta. All the analysis were performed blinded.

### Patient studies: healthy normal subjects and disease groups

Normal subjects (n=50, median age 47±17, 53% male) were recruited through advertising within the hospital, university and general practitioner surgeries. All normal subjects had no history or symptoms of cardiovascular disease or diabetes. Four subjects had been prescribed statin therapy for hypercholesterolaemia (primary cardiovascular prevention), but no other normal subject was taking any cardiovascular medication. All subjects had a normal blood pressure, 12 lead electrocardiogram and clinical CMR scan.

Patients (n=50) were prospectively recruited from tertiary clinical and research departments at the Heart Hospital or the National Amyloidosis Centre, Royal Free Hospital: [1] 12 patients with hypertrophic cardiomyopathy meeting the diagnostic criteria (average age 52±13, 60% male). [2] 18 patients with severe aortic stenosis waiting for aortic valve replacement (median age: 71±10, 72% male). [3] 20 patients with cardiac AL amyloidosis with disease proven by non-cardiac biopsy and cardiac involvement ascertained through echocardiography, supported by a Mayo clinic classification score of 2 or 3 (average age: 60±10, 75% male). Patients with atrial fibrillation or a contra-indication to contrast CMR examination were excluded from the study.

### Histological validation

24 patients with severe aortic stenosis listed for surgical aortic valve replacement were studied. An intraoperative deep myocardial biopsy (Tru-Cut needle) was taken in aortic stenosis. Samples were stained and analysed for CVF%, as previously described [9]. 6 patients were excluded from further analysis (2 patients had focal fibrosis detected as LGE in the basal septum; 2 patients had biopsies consisting solely of endocardial fibrosis; in 1 patient multibreath-hold T1 quantification was not performed because the patient was unable to breath-hold; 1 patient had a pulmonary oedema during the scan) leaving 18 patients.

### Reproducibility

For test:retest interstudy reproducibility, 10 normal subjects and 7 patients with amyloid underwent repeat scanning, one week apart. The analysis was carried out by a single observer blinded.

### Data analysis and statistics

Results were analysed using SPSS (Chicago, IL, USA, version 19). ECV values were found to be normally distributed in each group (Kolmogorov-Smirnov test, p>0.05 for each data set) and so expressed as mean ± standard deviation. We used Intraclass Correlation Coefficient (ICC) and Bland-Altman plot to compare ECV values from the two methods. Interstudy reproducibility for the CMR measurement of ECV was assessed by calculating the ICC and Bland Altman plots. To compare the squared difference of paired ECV measurements Wilcoxon signed rank test was used. A p value <0.05 was considered statistically significant.

## Results

### ECV comparison

**Figure 2:**
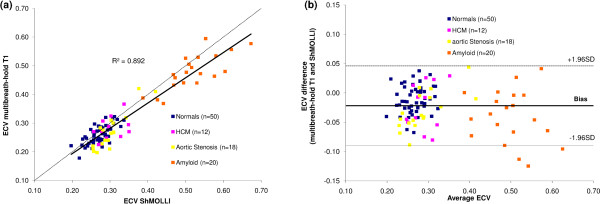
Sh-MOLLI and multibreath-hold ECV correlation in health and disease (panel a) and the same data plotted as a Bland-Altman analysis (panel b), showing little bias.

No patients failed ShMOLLI acquisition. 6% of patients were unable to perform all the 14 breath-holds required for the multibreath-hold technique. The mean ECV assessed using multibreath-hold T1 quantification and ShMOLLI T1 in normal subjects and disease groups is shown in Table 1. ECV by multibreath-hold T1 quantification and by ShMOLLI T1 mapping showed excellent correlation (r^2^=0.892) and agreement across disease groups (overall ICC 0.922, 95%CI 0.802 to 0.961, p<0.0001), Figure 2a, with little bias on Bland Altman (bias -2.2%, 95%CI -8.9% to 4.6%), Figure 2b.

**Table 1:** Mean ECV ± standard deviation assessed using multibreath-old T1 quantification and ShMOLLI T1 in normal subjects and disease groups

	**Multibreath-hold T1 ECV (%)**	**ShMOLLI ECV (%)**
Normal subjects (n=50)	26±3	27±3
HCM (n=12)	28±4	30±3
AS (n=18)	27±6	31±5
Amyloid (n=20)	48±6	52±7

### Histological validation

**Figure 3:**
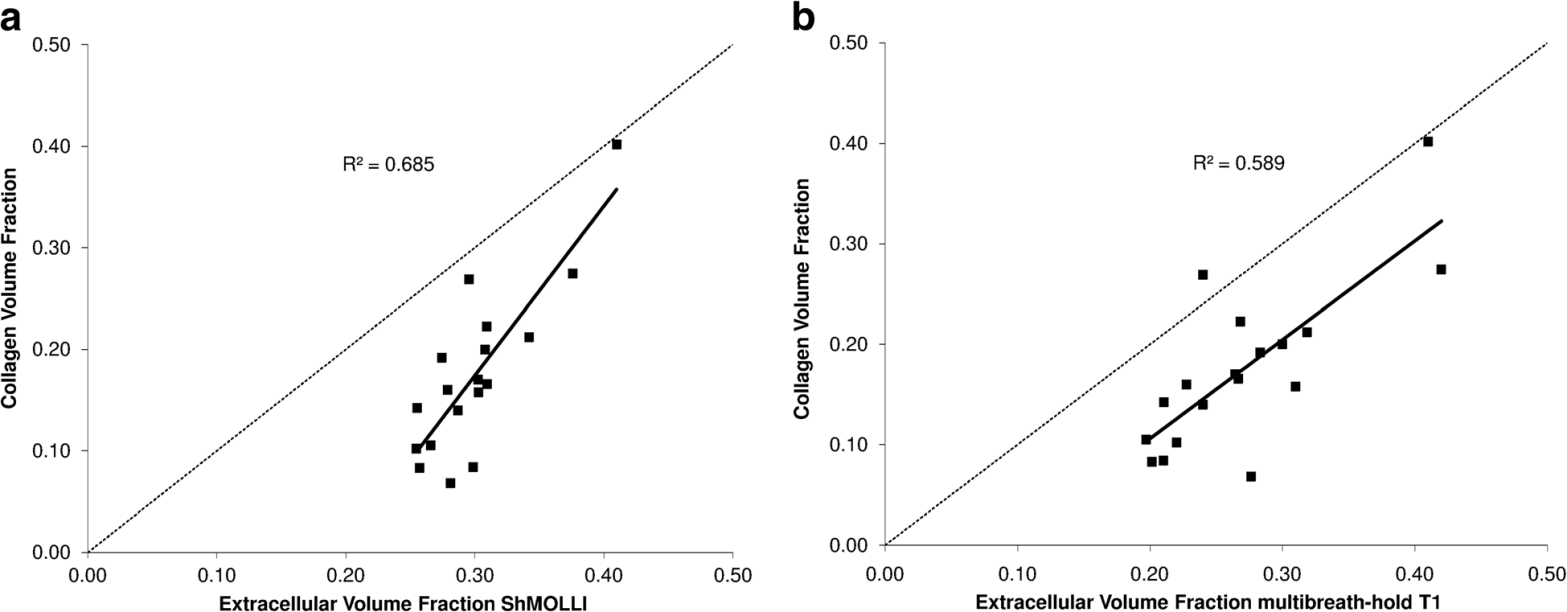
ECV against histological CVF% (n=18) by ShMOLLI (panel a) and multi-breath-hold (panel b).

All biopsies were uneventful. The mean histological CVF of the 18 biopsies was 18% ± 8% (range 7% to 40%). There was a strong correlation between histological CVF% and FLASH ECV (r^2^= 0.589) but this was stronger with ShMOLLI ECV (r^2^= 0.685) (Figure 3).

### Interstudy reproducibility

**Figure 4:**
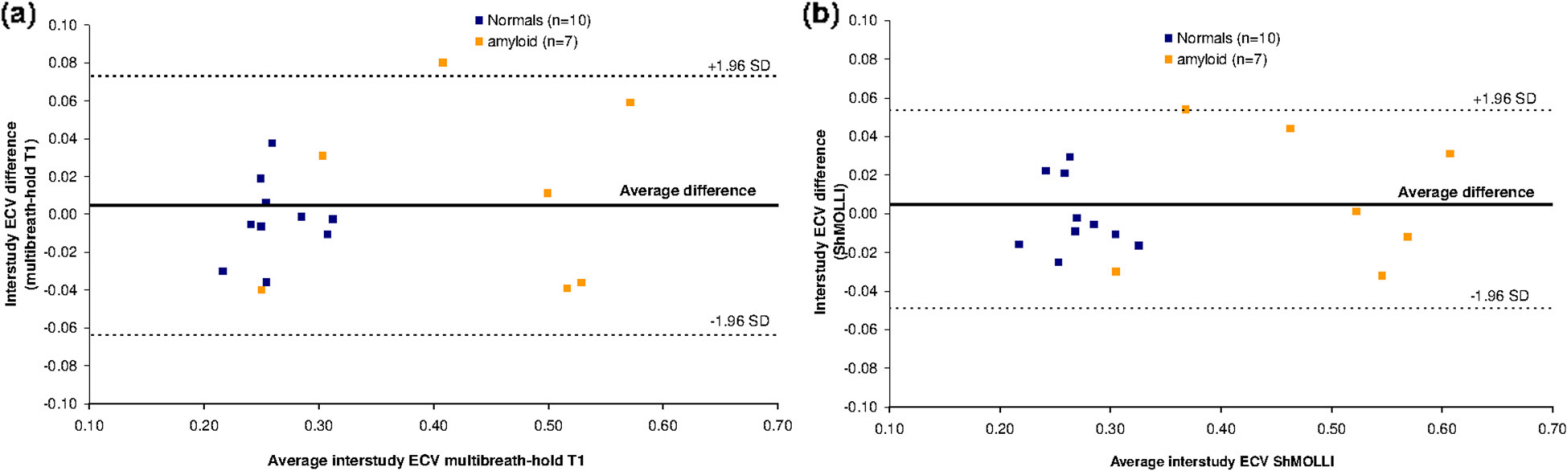
**Bland-Altman analysis to express the intrasequence reproducibility of the myocardial T1 values using FLASH-IR (panel a) and ShMOLLI (panel b) in normal subjects (blu square) and amyloid patients (orange square).** The limits of agreement are slightly narrower for ShMOLLI than multibreath-hold.

Inter-study reproducibility demonstrated that ShMOLLI ECV was slightly more reproducible than the multibreath-hold ECV, Figure 4, (ICC 982, 95%CI 0.951-0.994 versus ICC 962, 95%CI 0.897-0.987), with narrower confidence intervals (95%CI -4.9% to 5.4% versus 95%CI -6.4% to 7.3% respectively). Neither of these reached statistical significance however, P>0.2.

## Discussion

CMR ECV quantification requires accurate and rapid T1 relaxation time calculation. We have found that ECV quantification using ShMOLLI compared to multibreath-hold T1 quantification has a greater chance of technical success on all patients, less bias over a wide range of ECV measurements, a higher correlation with histological CVF% as well as better reproducibility. These four findings combine with key advantages of the mapping technique: a single breath-hold per T1 map, simple analysis and the potential for whole heart ECV quantification. Therefore, it is our opinion that T1 mapping technique ShMOLLI is the superior CMR technique for ECV quantification.

T1 mapping by means of the multi-breath-hold technique is one of the most commonly utilised methods for T1 quantification [9,10]. The main advantage of this technique is that it is not vendor specific and it has been heavily optimised by everyday clinical practice to obtain high resolution LGE images. However it also has a number of limitations. Firstly, in order to map a single cardiac slice, it requires the sequence to be run up to 9 times at increasing inversion times. The average breath-hold time for each sequence is 14 seconds (longer at slower heart rates). If the patient cannot hold their breath for the duration of the sequence, artefact will appear on any one of the images. This may also occur if the patient has an arrhythmia. As each slice takes so many breath-holds to map, this method can not be used clinically for whole heart mapping (i.e. of multiple cardiac slices). Once the images have been obtained, the offline post processing is laborious and the results require heart rate correction for the calculated T1 relaxations times [9].

We have found that ECV quantification using ShMOLLI improves on several practical aspects compared to multibreath-hold T1 quantification. ShMOLLI imaging is characterised by lower breath-hold failure rate, less bias over a wide range of ECV, a slightly higher correlation with histological collagen volume fraction and slightly better reproducibility, although it does not reach statistical significance. Shorter acquisition times and direct T1 map calculation on the scanner [12] ensures ShMOLLI is quicker to perform and analyse with a potential to provide whole heart ECV quantification. While currently ShMOLLI is vendor specific, its predecessor MOLLI exists for more than one platform [13].

Future techniques for T1 measurement are likely to be based on mapping. Further advances may include higher resolution imaging with reduction of partial volume effects, with that incorporate motion correction and improved capabilities for measuring longer T1s [14-17]. We preferred here to re-iterate the T1 map for maximum accuracy – a step that will likely be un-necessary in future refinements. Preliminary exploration is being made of combining the two T1 maps into an ECV map using further non-rigid registration [10].

This study has limitations. We have not presented the phantom work comparing ShMOLLI and FLASH-IR T1 estimation as this was primarily an ECV clinically-orientated paper with many of the likely confounders present only in-vivo and not detectable by phantom work. Histology correlations were lower previously described [9]. We believe this is primarily due to different population characteristics (the population examined here is older) and to a greater number of surgeons involved in the biopsy arm of this study (with associated reduced homogeneity of samples). Finally, FLASH-IR was compared to a single T1 mapping technique, ShMOLLI. MOLLI,its variants and other T1 mapping techniques such as SACHA [18] were not considered in this study.

As ECV quantification experience increases, it is likely that technology will advance to become more precise, accurate and automated. Our study represents the first such paper and demonstrates that single breath-hold ShMOLLI T1 mapping can quantify ECV by EQ-CMR across the spectrum of interstitial expansion, and that it is clinically more straightforward with improvements in reproducibility and histological correlation when compared to the older multibreath-hold FLASH techniques.

## Conclusion

ECV quantification by single breath-hold ShMOLLI T1 mapping can measure ECV by EQCMR across the spectrum of interstitial expansion. It is procedurally better tolerated, slightly more reproducible and better correlates with histology compared to the older multibreathhold FLASH techniques.

## Competing interests

The authors declare that they have no competing interests.

## Authors’ contribution

MF: led the study; co-ordinated analysis; lead writer of manuscript. SKW, SMB, DMS, VM each contributed a patient group along with the respective analysis. SKP & SN: technical support for T1 mapping sequence/ShMOLLI development, analysis of T1 maps. ASF: blind analysis of histological data. NR: co-ordinated the surgical support and biopsy. JCM: concept and design of study. All authors read and approved the final manuscript.
